# Lead Exposure Assessment among Pregnant Women, Newborns, and Children: Case Study from Karachi, Pakistan

**DOI:** 10.3390/ijerph14040413

**Published:** 2017-04-13

**Authors:** Zafar Fatmi, Ambreen Sahito, Akihiko Ikegami, Atsuko Mizuno, Xiaoyi Cui, Nathan Mise, Mai Takagi, Yayoi Kobayashi, Fujio Kayama

**Affiliations:** 1 Department of Community Health Sciences, Aga Khan University, Karachi 74800, Pakistan; zafar.fatmi@aku.edu (Z.F.); ambreensahito@gmail.com (A.S.); 2 Department of Environmental and Preventive Medicine, School of Medicine, Jichi Medical University, Shimotsuke 329-0498, Japan; axi21@jichi.ac.jp (A.I.); xiaoyicui0508@gmail.com (X.C.); nmise@jichi.ac.jp (N.M.); 3 Department of Pharmacology, School of Medicine, Jichi Medical University, Shimotsuke 329-0498, Japan; aaamiz@jichi.ac.jp; 4 Center for Health and Environmental Risk Research, National Institute for Environmental Studies, Tsukuba, Ibaraki 305-0053, Japan; takagi.mai@nies.go.jp (M.T.); kobayashi.yayoi@nies.go.jp (Y.K.)

**Keywords:** Lead (Pb), surma, pregnant women, newborn, children, Pakistan

## Abstract

Lead (Pb) in petrol has been banned in developed countries. Despite the control of Pb in petrol since 2001, high levels were reported in the blood of pregnant women and children in Pakistan. However, the identification of sources of Pb has been elusive due to its pervasiveness. In this study, we assessed the lead intake of pregnant women and one- to three-year-old children from food, water, house dust, respirable dust, and soil. In addition, we completed the fingerprinting of the Pb isotopic ratios (LIR) of petrol and secondary sources (food, house-dust, respirable dust, soil, *surma* (eye cosmetics)) of exposure within the blood of pregnant women, newborns, and children. Eight families, with high (~50 μg/dL), medium (~20 μg/dL), and low blood levels (~10 μg/dL), were selected from 60 families. The main sources of exposure to lead for children were food and house-dust, and those for pregnant women were soil, respirable dust, and food. LIR was determined by inductively coupled plasma quadrupole mass spectrometry (ICP-QMS) with a two sigma uncertainty of ±0.03%. The LIR of mothers and newborns was similar. In contrast, *surma*, and to a larger extent petrol, exhibited a negligible contribution to both the child’s and mother’s blood Pb. Household wet-mopping could be effective in reducing Pb exposure. This intake assessment could be replicated for other developing countries to identify sources of lead and the burden of lead exposure in the population.

## 1. Introduction

Lead (Pb) exposure causes an estimated 0.6% of the global burden of disease, predominantly occurring in developing countries [1]. Every year, 143,000 deaths and 600,000 new cases of intellectual disabilities occur due to lead exposure [1]. Lead may affect the health of an individual by injuring the kidney, liver, and the haematological and neurological systems [2,3,4].

Lead has contaminated the environment including food, soil, water, and air, mainly through its usage in petrol. The decrease in the usage of lead in primary sources such as petrol has substantially reduced the population exposure. However, lead exposure is still excessive in several developing countries [5,6,7,8,9]. The reduction of lead in primary sources has also enhanced the importance to ascertain the secondary sources of lead. Nonetheless, due to the pervasive use of lead, it is difficult to determine the major sources of lead exposure in these environments.

One of the modern methods used to ascertain the sources of lead exposure is lead isotope ratio (LIR) analysis. In the environment, lead exists as four main isotopes: ^204^Pb, ^206^Pb ^207^Pb, and ^208^Pb. The most common is ^208^Pb (52%), followed by ^206^Pb (24%), ^207^Pb (23%), and ^204^Pb (1%). Of these, three isotopes (^206^Pb, ^207^Pb, and ^208^Pb) are produced by the radioactive decay of ^238^U, ^235^U, and ^232^Th, respectively. ^204^Pb is the only primordial stable isotope. Thus, the abundance of Pb isotopes in a sample depends on concentration of U, Th, and primordial Pb in the source and the time elapsed since their formation [10,11]. The composition of Pb isotopes is commonly expressed as a ratio. The ratios ^206^Pb/^204^Pb, ^206^Pb/^207^Pb, ^208^Pb/^206^Pb, ^207^Pb/^204^Pb, and ^208^Pb/^204^Pb are the most preferred because these can be determined more accurately. The isotopic compositions of Pb are not significantly affected by physico-chemical fractionation processes. Therefore, Pb isotopes are considered a proficient tool for determining the sources and pathways of Pb exposure [11].

Although few in number, all studies have reported high blood lead levels in Pakistan since 1989 [12,13,14,15,16,17,18]. Comparatively higher blood lead levels were reported from the megacity Karachi (range 7.2–38.2 µg/dL) and Islamabad with less dense traffic (3.22–2.3 µg/dL) [12,13,14,15,16,17,18]. Moreover, higher blood lead levels have also been reported among children of industrial workers [19].

Therefore, the current study has empirically assessed the potential sources of lead exposure among pregnant women, newborns, and young children (one to three- years old) living in the same households in the city of Karachi, Pakistan. A comparison of lead of isotope ratios (LIR) among pregnant women’s blood was done to estimate the exposure among newborns. The investigation of exposure among one to three year olds reflected environmental exposure of lead, particularly due to sources within the household. We analysed the common sources, including food (three-day food duplicate samples), petrol, *surma* (eye cosmetics), soil, house dust, and respirable dust in the households. The study determined the percentage uptake of lead using LIR and the in-vitro bioaccesibility (source apportionment) for each source for pregnant women and the one- to three-year-old children.

## 2. Materials and Methods

This cross-sectional study was conducted during August 2014 to November 2015 in Karachi, which is the largest megacity of Pakistan with a population >20 million. Consent was taken from multiparous pregnant women visiting a tertiary care hospital (Qatar Hospital, Orangi Town, Pakistan) for prenatal care, who had at least one living child between one to three years of age and who had been a resident of the city of Karachi for the past four years.

A sample of peripheral venous blood from pregnant women and cord blood from the newborn were taken at delivery. Newborn clothes were provided as an incentive. Of the 66 women who came for delivery, 52 also agreed to give the blood of their one- to three-year-old child and were followed up at their homes after one month of delivery. We visited 66 homes for three consecutive days to collect several samples from the household for the determination of lead exposure for the women and child of the same family. These samples included separate food duplicate samples for the women and one- to three-year-old children, and those of house-dust, respirable dust (air sample), drinking water, and soil around the house. Additionally, we collected petrol and engine lubricant samples from neighborhood gas stations. These samples were shipped to Japan for further analysis. Eight families were selected based on the blood lead levels of the pregnant women for a more detailed analysis: two with high levels (~50 µg/dL), two with medium levels (~20 µg/dL), and four with low levels (~10 µg/dL).

### 2.1. Sample Collection and Preparation

The details of the samples and sampling strategy are as follows.

#### 2.1.1. Blood Samples and Preparation

One mL of blood from the pregnant women, umbilical cord, and children was digested separately with 2 mL of nitric acid Ultrapur-100 (Kanto Chemical Co., Inc., Tokyo, Japan) in a microwave digestion system TOPwave (Analytik Jena Japan Co., Ltd., Kanagawa, Japan), according to the instruction manual, and were analyzed by inductive coupled plasma–mass spectrometry (ICP-MS).

#### 2.1.2. Food Sample Collection and Preparation

Three-day, two weekdays, and one weekend food duplicate samples (i.e., the same amount of food and water, including snacks eaten) were obtained from the women and children. Nominal money was paid to the households for obtaining the food samples. The samples were collected in lead-free plastic bags or a stainless steel box (SUS302), and all liquid food and drinking water samples were individually stored in polypropylene bottles for each meal. The food items were also self-recorded by the women in a food diary for confirmation.

The entire three-day food samples were ground to make a paste by a food processor (Magimix Compact 3200XL; Magimix UK Ltd., Surrey, UK). The entire three-day sample was then mixed into a pooled sample for each subject. If the food was too solid for grinding, then a measured amount of deionized water was added. The ground food was further homogenized using a Polytron homogenizer PT10-35 GT (KINEMATICA AG., Luzern, Switzerland). The homogenized samples for the women and children were kept separately in plastic tubes. Homogenized food samples (2 g) were digested with 5 mL of nitric acid Ultrapur-100 and 1 mL of hydrogen peroxide for atomic absorption spectrometry (Wako Pure Chemical Industries, Ltd., Osaka, Japan) using the microwave digestion system TOPwave. ICP-MS analysis was conducted by Japan Food Research Laboratories (JFRL; Tokyo, Japan), which is certified by ISO9001, ISO/IEC 17025 ISO9001, ISO/IEC 17025, and JAS, using Agilent 7500ce (Agilent Technologies Japan, Ltd., Tokyo, Japan),

#### 2.1.3. Water Sample Collection and Preparation

Morning tap drinking water samples were collected in 25 mL centrifuge tubes (AGC TECHNO GLASS Co., Ltd., Shizuoka, Japan). In the case where more than one source of drinking was used, the most commonly used was sampled. Water samples were filtered with a 0.45 μm cellulose acetate disk filter MILLEX-HA 33 mm diameter (Millipore Corporation, Billerica, MA, USA) and 1/100 volume of nitric acid was added to the filtered samples.

#### 2.1.4. House Dust Collection and Preparation

Dust was obtained in bagless vacuum cleaners (Dyson DC50 upright vacuum cleaner; Dyson Inc., Chicago, IL, USA) during routine cleaning from the places in the house where the children spent the most amount of time.

The dust was then sieved through an opening size of 100 µm (Tokyo Screen Co., Ltd., Tokyo, Japan). The hairs and fibrous materials which passed through the sieve were manually removed. The dust samples were dried at 60 °C overnight in an oven, and kept in separate plastic bags in a cool and dry environment away from sunlight and fumes.

#### 2.1.5. Particulate Matter (Respirable Dust) Collection and Preparation

Particulate matter of PM_4_ (median aerodynamic diameter 4 μm, 50% cut) was collected for 24-h from each household. PM_4_ was considered appropriate for the determination of lead concentration in the air. The low volume air sampler with the dust separator model C-30 (Sibata Scientific Technology Ltd., Saitama, Japan), with a suction flow rate of 9.6 L/min, at a height of 50 cm above the floor (to simulate child’s respiratory zone), was used. The sampler was placed in the room where the children spent most of their time. Dust was collected on two glass-fiber filters of 55 mm diameter as supplied from the vendor for conformity with the separator. Filters with collected dust were kept in separate plastic bags in a cool and dry environment away from sunlight and fumes before analysis.

#### 2.1.6. Petrol, Engine Lubricant and Surma (Eye Cosmetics) Sample Collection and Preparation

A total of seven samples (six for petrol and one for engine lubricant) were obtained from gasoline stations from the Orangi town neighborhood, from where all other household samples were collected. Also, several samples of surma/kohl (eye cosmetics) were bought from the open market in Karachi. These samples were kept in lead-free containers. Pb(C_2_H_5_)_4_ and another alkyl lead in the samples were converted with trace metal grade concentrated hydrochloric acid (Wako Pure Chemical Industries, Ltd., Osaka, Japan) to PbCl_2_, by mixing it overnight at room temperature. All of the lead in the mixture was extracted by ultrapure water for analysis.

#### 2.1.7. Soil Collection

Soil samples were collected in the vicinity of each participating family. The first soil samples were discarded at Japanese Quarantine Office on arrival. We then collected more soil samples in the same places and prepared the samples using acidic extraction fluid in Karachi and the sample fluids were transferred to Japan.

### 2.2. Extraction of Bioaccessible Lead

The extraction of bioaccessible lead was carried out using the standard operating procedure for an in vitro bioaccessibility (IVBA) assay for lead in soil [USEPA 2012]. This method is validated by the United States Environmental Protection Agency (USEPA) for an in vitro assay used for estimating lead relative bioavailability (RBA) in environmental media (soil, dust, food, etc.).

The extraction fluid used was 0.4 M glycine (free base, reagent grade glycine in deionized water), adjusted to a pH of 1.50 ± 0.05 using trace metal grade concentrated hydrochloric acid. Soil samples (200 mg) were mixed with the extraction fluid to a solid-to-fluid ratio of 1/100 (mass per unit volume) in a 25 mL lead-free tube. Samples were extracted at 37 °C, at 30 rpm in a BR-40LF bio-shaker (TAITEC Corporation, Saitama, Japan) for one hour, ensuring that the pH was maintained at 1.5 ± 0.5. The extracts were filtered with a 0.45 μm cellulose acetate disk filter (33 mm diameter) and the filtered samples were stored at 4 °C. The samples were analyzed by ICP-MS Agilent 7500cx in NIES (Agilent Technologies Japan, Ltd., Tokyo, Japan).

### 2.3. Analysis to Determine the Lead Concentration

The lead concentrations in blood, food, water, petrol, and engine lubricant, as well as the extraction fluids for bio-accessible lead from environmental media (food, house dust, respirable dust and soil), were determined using inductively coupled plasma-mass spectrometry (ICP-MS). The ICP-MS Agilent 7500cx (Agilent Technologies Japan, Ltd., Tokyo, Japan) method was performed in The National Institute for Environmental Studies (NIES), Japan.

The measurement for lead was carried out by the calibration curve method using a lead standard solution (Wako Pure Chemical Industries, Ltd., Osaka, Japan) and a thallium standard solution (Wako Pure Chemical Industries, Ltd., Osaka, Japan) for an internal standard. The lower limit detection of lead was 0.001 ng/mL (ppb).

The test for quality control was performed by using commercial reference samples: National Institute of Standards and Technology (NIST) Standard Reference Material (SRM) 995c, Toxic Metals in Caprine Blood (NIST, Gaithersburg, MD, USA) for blood analysis; National Metrology Institute of Japan (NMIJ, Tsukuba, Japan) Certificated Reference Materials (CRM) 7202-b, Trace Elements in River Water (Elevated Level) (NMIJ, Tsukuba, Japan) for water analysis. The recovery of lead for the blood and water analysis methods was 95.7%, and 92.9%, respectively.

### 2.4. EDXRF Analysis for House Dust and Respirable Dust

Energy dispersive X-ray fluorescence spectrometry (EDXRF) was conducted by the Industrial Technology Center of Tochigi Prefecture, using an JSX-3100RII element analyzer (JEOL, Tokyo, Japan) to determine the lead concentrations of house dust and respirable dust.

For analysis, house dust was placed in a specific plastic cup with thin film sample supports of PROLENE 4.0 microns (Chemplex Industries, Inc., Palm City, FL, USA) and it was pressed by hand using a pestle. House dust samples were analyzed for 240 s (live time) under an air-condition using an X-ray lamp voltage of 50 kV, an auto lamp current, a 7 mm collimator, and a Pb filter. The measurement for lead was carried out by the calibration curve method equipped in the instrument. Samples for the calibration curve were prepared by cellulose, powder (Nacalai Tesque, Inc., Kyoto, Japan), and NIST SRM 2583.

For analysis, deposited respirable dust on the filter was placed on the measurement stage with the PROLENE film to prevent the contamination of the detecting element. Respirable dust samples were analyzed for 600 s (live time) under the same condition as for the house dust analysis. The measurement for lead was carried out by the calibration curve method. The standard filters for the calibration curve were prepared by the droplet method.

### 2.5. Lead Isotope Ratios Analysis

The acid digested solution of blood samples, food, and the extraction of bioaccessible lead from environmental media (house dust, respirable dust, soil, surma, petrol and engine lubricant), was analyzed for a comparison of the lead isotope ratios (LIR). Measurements of the LIR ^207^Pb/^206^Pb versus ^208^Pb/^206^Pb were performed using ICP-QMS Agilent 7500cx in NIES. The instrumental conditions of ICP-QMS for LIR analysis are given in Table 1 (for details see Takagi et al.) [20]. The National Institute of Standards and Technology (NIST) Standard Reference Material (SRM) 981, Common Lead Isotopic Standard, was used to correct for mass discrimination. The typical within-run RSD of the isotope ratio measurement of NIES SRM 981 was around 0.3% for both ^207^Pb/^206^Pb and ^208^Pb/^206^Pb. The extraction of NIST SRM 2583 was analysed for every ICP-QMS measurement for quality control. The value (n = 5) was 0.822 ± 0.002 for ^207^Pb/^206^Pb and 2.027 ± 0.003 for ^208^Pb/^206^Pb, which agreed with the value measured by the ICP-MS multi-collector (unpublished data, 0.8241 ± 0.0000 for ^207^Pb/^206^Pb and 2.0279 ± 0.0001 for ^208^Pb/^206^Pb).

The mean and standard error for LIR of ^207^Pb/^206^Pb and ^208^Pb/^206^Pb were separately determined for the blood of pregnant women, newborns, and children, as well as for all of the environmental media and food samples of pregnant women and children.

### 2.6. Lead Contamination Tests from Cooking Utensils

We also measured the lead concentration for raw (pre-cooked) and cooked food for common food items to ascertain the contribution of cooking utensils for increasing the lead levels in the food. Five different types of utensil, including commonly used alloys, steel, iron, non-stick utensils, and microwaves, were used for cooking the same food. Three common foods items including lentils (daal), potatoes, and chicken were assessed. The raw and cooked food items were processed in the same manner as the food duplicate samples and were measured for lead contents (Appendix Table A2).

### 2.7. Calculation of Lead Uptake and Statistical Analysis

To compare the contribution of various lead intake sources, a calculation for the lead uptake from food, water, house-dust, respirable dust (PM_4_), and soil [in µg/kg body weight/week] was conducted with the following calculation formula, based on the USEPA Exposure Factors Handbook 2011 edition [18].

(1) Food=[C×DI÷BW]×7

(2) Water=[C×DI÷BW]×7

(3) House dust=[C×IngR÷BW]×7

(4) Respirable dust=[C×InhR÷BW]×7

(5) Soil=[C×IngR÷BW]×7

where *C* is the concentration of lead in the respective media [food, µg/g; water, µg/mL; house dust, µg/g; respirable dust, µg/m^3^; soil, µg/g]; DI is the calculated daily intake of food (mg/day) and water (mL/day); IngR is the ingestion rate of house dust (for adults: 30 mg/day; child (1–6 years): 60 mg/day) and for soil (for adults: 20 mg/day; child (1–<6 years): 50 mg/day); InhR is the inhalation rate of respirable dust (for adults of normal weight between 23–<30 years, pregnancy 22nd week): 21.4 m^3^/day and child (2–< 3 years) 8.9 m^3^/day); BW is the body weight, kg; multiplied by seven to convert it into a weekly dose. The daily intake of food and water was calculated by the weighed value at sample preparation. For all of the ingestion and breathing rates, we used the data from the USEPA Exposure Factors Handbook 2011 edition [18].

Environmental media including house-dust, respirable dust, soil, petrol, surma, and water, as well as food, were extracted as bioaccessible lead. To assess the contributions of ingestion of these potential environmental lead sources, the calculation methods were used.

The study was given approval by the Ethics Review Committee of Aga Khan University and the Institutional Review Board of Jichi Medical University, Japan.

## 3. Results

The mean blood lead levels of the overall sample for pregnant women, one- to three-year-old children, and umbilical cord blood are provided in Table 2.

Among the selected eight households, based on the mothers’ blood lead level, families (A–H) were categorized into three groups: two families with a high blood lead group [~50 µg/dL], two for a medium blood lead group [~20 µg/dL], and four families for a low blood lead group [~10 µg/dL]. The cord blood lead levels were closer to the blood lead levels of pregnant women (~80%, ranged 46%–118%) (Table 3).

The lead levels of the pregnant women’s blood were highly correlated (spearman’s ρ) with cord blood lead levels (*r_s_* = 0.86; *p* = 0.007). The cord blood levels were also highly correlated with the one- to three-year-old children (*r_s_* = 0.86; *p* = 0.007) (Appendix Table A1).

The lead content levels of three common food items (i.e., chicken as meat, lentils, and potato as vegetables) were similar before and after cooking using four different utensils, suggesting a minimal contribution of utensils for increasing the lead content in the food. However, the lead concentrations for all food items were lower than the controlled limits (Appendix Table A2).

The lead concentrations of the petrol and engine lubricant from gas stations in the neighborhood of the study participants ranged from 0.013 to 0.083 mg/L, well below the control levels of less than 20 mg/L (Appendix Table A3).

**Figure 1:**
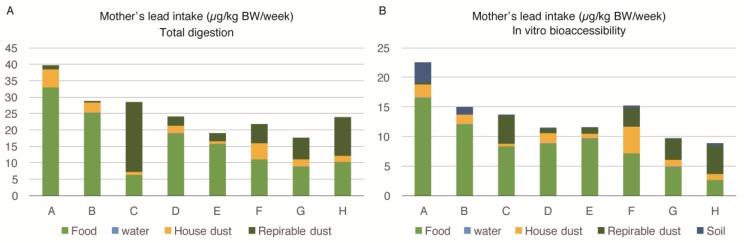
Lead intake by multiple sources among pregnant women of eight families (A–H) in Karachi, Pakistan: (**A**) Lead intake (total acid digestion) from sources in µg/kg BW/week; (**B**) Lead intake measured as in-vitro bioaccessible lead in µg/kg/week.

Figure 1A shows the mothers’ lead intake from food, water, house dust, and respirable dust, calculated by the equation in Section 2.7. In this calculation, we used the lead values of house dust and respirable dust measured by *EDXRF analysis* and food and water determined by ICP-MS after total acid digestion by the microwave. As the soil in Karachi could not be transported to Japan, the soil data is missing in this figure. Figure 1B depicts a mother’s in-vitro bioaccesibility (IVBA). All of the bioaccessible lead values used were measured after the extraction described in Section 2.2. The body intake of lead among pregnant women from different families (A to H) ranged from 8.9 to 22.6 µg/kg body weight/week. The food was the most important source of lead intake among pregnant women. The IVBA of lead from food ranged between 29%–83% (mean = 62.37%). The contribution of lead by food was higher for families with a higher exposure to lead.

The second most important source of lead exposure among pregnant women was respirable dust (PM_4_) intake, which ranged from 2% to 75% (mean = 27.12%), while the IVBA ranged from 0% to 54% (mean = 20.37%). The percentage contribution of lead by respirable dust was higher for families exposed to lower levels of lead. House dust was also an important source; however, water was contributing a negligible amount of lead intake among pregnant women.

**Figure 2:**
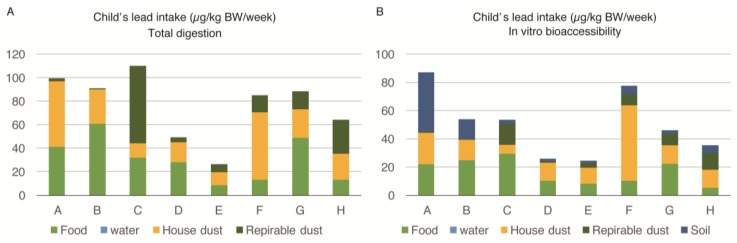
Lead intake (in-vitro bioaccessiblity) from multiple sources among one- to three-year-old children of eight families (A–H) in Karachi, Pakistan: (**A**) Lead intake from sources in µg/kg BW/week; (**B**) Percentage contribution from each source.

The lead intake by various sources among the one- to three-year-old child of the family is described in Figure 2A,B. The intake of lead seen for each child was almost three times higher compared to that of pregnant women, and ranged from 24.4 to 87.3 µg/kg body weight/week. Both the food and house-dust equally contributed to the body burden of each child’s lead levels. The proportion of lead intake by food ranged between 15%–67% (mean = 39.75%), while it ranged between 11%–68% (mean = 38.12%) due to house-dust. The IVBA of lead from food ranged between 13%–55% (mean = 34.50%), while it ranged between 12%–69% (mean = 36.75%) due to house-dust. Respirable dust (PM_4_) and soil were also important sources of exposure for one- to three-year-old children, as opposed to pregnant women. However, lead from water was contributing a negligible amount to the body burden of lead in the young child similar to the pregnant women. There were no marked differences in the sources of exposure (percentage contribution of lead) for young children among families exposed to high and low lead levels. The children of all selected families were similarly exposed to lead from food, house dust, respirable dust, and soil.

The bioaccessibility of lead in different sources was calculated in the data presented in Figure 1A,B and Figure 2A,B (Appendix Table A4). The average values show that approximately 60% of the lead contents were extracted from food and house dust samples, but a lower percentage of lead originated from the respirable dust.

**Figure 3:**
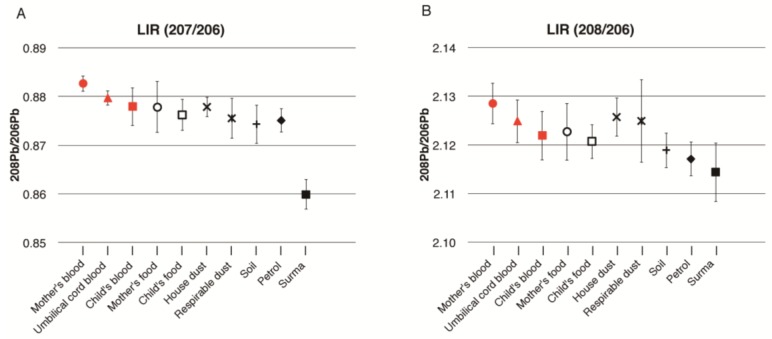
Lead isotopes ratios (LIR) for eight families (combined) in Karachi: (**A**) LIR 207/206; (**B**) LIR 208/207. Legends: Mother’s blood (●), Umbilical cord blood (▲), Child blood (■), House dust (☓), Respirable dust (✳), Soil (+), Mother food (о), Child food (□), Petrol (◆), Surma (■).

Figure 3 is a graphical representation of the mean LIR of mother’s, cord, and child’s blood, and the environmental samples for all eight families. The LIR of pregnant women, cord blood, and children’s blood were very similar based on their error bars. These LIRs were also close to those of house dust and respirable dust. However, the LIR of 208/206 of the children in Figure 3B is lower than the values seen for the mothers’ and cord blood, which is rather close to the value of soil. The LIR of petrol was not related to the LIR of the blood of pregnant women or cord blood, but slightly overlapped with that of the young children’s blood. Additionally, the LIR of petrol is rather close to that of soil. Moreover, the LIR of surma had no similarity to pregnant women, cord blood, or the children’s blood lead level.

## 4. Discussion

Recent studies in Pakistan have shown high blood lead levels in the vulnerable population, including newborns and children [12,13,14]. The current study empirically ascertained the main sources of Pb exposure and the proportion contribution for blood from potential sources using IVBA extraction among pregnant women/newborns and the one- to three-year-old children in the megacity of Karachi, Pakistan. In this regard, few studies are available regarding the source apportionment of lead exposure using food duplicate studies and other potential sources from developing countries [21].

Besides validating the findings of high lead exposure among this population, the study identified that food, house-dust, and respirable dust were the main sources contributing to the lead level in the blood of pregnant women, and food and house-dust contributed the most to the lead level seen among young children. The contribution of dust to the blood lead level is most critical for children aged one to three years, typically with the highest lead levels and greater hand-to-mouth activity [22]. For the individuals older than four years, hand-to-mouth activity is minimal and diet assumes a greater importance as a source of lead [23].

Previous available studies in Pakistan had limitations, as these were purely epidemiological in nature and had identified behavioral and subjective factors [12,13]. Some of the previous investigations had also misled the researchers and policy makers. For example, water has been implicated as a major source of exposure for taking countermeasures against lead [24]. This study clearly identified that water was not a major source of exposure among the pregnant women and children. Similarly, surma (eye cosmetic) was not a major contributor to the body burden of lead. A previous study has implicated surma as a major source of lead exposure in the same population [12].

Studies used to identify lead sources are so far based on behavioural studies and the determination of lead levels in samples of petrol, paint, dust, soil, and water, separately. These measurements have been made without objectively linking them to determine the proportion contribution of these sources for blood lead levels. A study in several geographically different locations in Karachi suggested that high blood lead levels were related to vicinity to the main street and intersection, *surma*/*kohl use* (eye cosmetic), father’s occupational lead exposure, a parent’s illiteracy, and a child’s habit of hand-to-mouth activity [18]. Another study conducted in Karachi city found that umbilical cord blood levels were higher among mothers living in houses with windows open, those using *surma* daily, and in households where the mothers took no calcium or less iron supplements during pregnancy [12]. In one study, water has also been found as a major source of lead in Karachi [24]. All of these studies point to one or the other source of lead exposure. However, the information from these studies does not provide the exposure contribution from sources for pregnant women, newborns, and small children.

This study is the first food duplicate study in Pakistan and provides information about the oral intake of lead in food. Few studies have conducted the measurement of lead exposure through food. Since the implementation of unleaded gasoline in developed and many developing countries, food may be considered as a major source of secondary exposure. However, due to the unavailability of reliable methods and laboratories, it has not been studied, particularly in developing countries. The proportion of bioaccessible lead from multiple sources and source apportionment using LIR were estimated for pregnant women and young children in Karachi, to identify the important contributors of lead in these vulnerable populations. Food, house-dust and respirable dust were identified as major sources of exposure among pregnant women. Besides food, house dust was identified to contribute to blood lead levels among young children in Pakistan. This investigation informs that regular wet-mopping in the households could be an important intervention for the prevention of exposure to lead. Also, further investigations are needed to identify the contamination sources of food and major foods contributing to lead exposure in this population.

We used isotopic analysis by ICP-QMS validated by a ICP-MS multi-collector. Our analysis of ILR was not precise enough to determine the percentage contribution of lead from individual sources. However, LIR of pregnant women’s blood and cord blood were closely related in most families and the child’s blood was more closely related with current environmental sources of exposure such as food, house-dust, and respirable dust. The LIR of petrol (largely) and *surma* (particularly) was distinct from the blood LIR of pregnant women, newborns, and young children in most families, indicating that these are not the primary major sources of exposure.

Pregnant women’s LIR was relatively higher and distinct from all other current sources of lead exposure, suggesting past exposure and the mobilization of lead deposited in bone tissue. Alternatively, pregnant women’s exposure might relate to some other environmental sources which have not been studied in this investigation. However, a strong relation of a newborn’s and child’s blood LIR with current sources clearly indicate that the lead level of pregnant women could only be due to past exposure. The lead deposits in the bone tissues remain in constant exchange with blood and that might be a larger contributor to pregnant women’s blood lead levels, particularly during pregnancy and breast feeding [25,26].

The uptake of lead per body weight, as determined by IVBA, by young children, was almost three times higher compared to pregnant women (Figure 1B and Figure 2B). This is an alarming level of exposure for the vulnerable population. It means that the exposure of young children after they were born tended to increase and would have severe detrimental effects on their developing brains. A marked improvement in the overall environment of the children is required in Pakistan and developing countries, to reduce lead exposure. Household cleaning practices and behavioral interventions are needed to decrease the lead exposure among young children in the households in Pakistan. Wet-mopping of household could be a key intervention to reduce lead exposure.

We further investigated the main source of exposure, i.e., food, for possible sources of contamination. First, we investigated lead contamination through cooking with several cooking utensils in a laboratory in Aga Khan University. The increment of lead after cooking was negligible and the lead concentrations in the food ingredients examined were generally low (Appendix Table A2). It suggests that food samples might be contaminated with lead from house dust during cooking in the kitchen, and during the sampling and processing process. Manton et al. [27] revealed that in the LIR study in Omaha and Nebraska during the period of 1990 to 1997, most of the dietary collection contained a large component of house dust.

Therefore, we suggest that, first, a more systematic surveillance for lead contamination in food and the environment is required in Pakistan. Second, we must delineate possible contamination sources during agricultural and animal farming practices and the processing of various food items in Pakistan.

The currently available petrol contains lead levels much lower than the recommended guidelines (<20 mg/L). It is evident that current automobile exhaust gas is not a major lead contamination source. We could not obtain gasoline or alkyl lead used in the past. However, we speculate that similar alkyl lead was added to gasoline in the past, which produced ubiquitous lead contamination that is sustained in the environment. This is supported by studies conducted in western countries which show that emitted lead remains a source of exposure for a longer duration, maybe decades [27]. The lead content in petrol has gradually decreased in Pakistan from 1.5–2.0 g/L in 1991, to 0.4 g/L in 1993–1996, and then to 0.36 g/L in 1999. Lead has been controlled in gasoline sources since 2001 to less than 0.02 g/L [28,29]. Nonetheless, there are some unanswered questions regarding whether food was contaminated with lead by the absorption from farmland soil or deposited from fallout dusts during transportation and cooking. This needs further investigation.

There were certain limitations in this analysis which need to be considered. As the samples were collected from one megacity, the study findings can only be applied to this city. However, Karachi is a megacity where approximately 10% of the population of Pakistan resides. Also, being the main harbor of the country, most of the petrol and food are processed and transported up-country from Karachi, so we consider that a similar LIR would be prevalent in other parts of the country. Due to the high cost and time required to conduct the laboratory analysis, for several matrices of triad (pregnant women, newborns, and young children), as well as for four isotopes, we limited the analysis to eight families. However, the samples were selected from a larger study based on the blood lead levels among pregnant women. The samples chosen were from both high and low levels of lead exposure among the same population exposure range.

Nevertheless, the study methodology can be used for determining the sources of lead exposure in similar situations. To the best of our knowledge, it is among the first few studies of this nature which has comprehensively determined the source apportionment and utilized LIR analysis to compare patterns of Pb exposure in blood specimens, food duplicates, and environmental samples in a developing country. The information would provide management strategies for public health action.

The study capitalized on the strong collaboration between developing and developed country and we feel that this has been an important strength of this study. The methodology required several sophisticated advanced analyses, which are generally not available in a developing country like Pakistan. The limited capacity has been the major limitations for such studies to be replicated in developing countries.

## 5. Conclusions

High levels of blood lead were present among pregnant mothers and young children and may induce adverse developmental effects in the newborns and young children. Food, house-dust, and respirable dust among pregnant women and young children were the main contributor of blood Pb in this population. Surma, and to a large extent petrol, are not major contributors of the blood lead levels of mothers and children in Pakistan. Behavioral interventions such as wet-mopping and clean cooking practices may help to control the lead exposure among this population. Therefore, a surveillance of lead contamination is urgently needed to devise countermeasures to reduce environmental lead contaminations.

## Figures and Tables

**Table 1:** Instrumental conditions of ICP-QMS for lead isotope ratio (LIR) analysis.

Parameters	Conditions
RF (W)	1600
Plasma gas (Ar) flow rate/L·min^−1^	15.0
Carrier gas (Ar) flow rate/L·min^−1^	0.90
Auxiliary gas (Ar) flow rate/L·min^−1^	0.90
Makeup gas (Ar) flow rate/L·min^−1^	0.20
Sample uptake rate/rps	0.1
Acquisition time/point·mass^−1^	3
Dwell time/s·points^−1^	1
Integration time/s·points^−1^	3
Number of measurement/times	10
Monitor mass/m·z^−1^	206, 207, 208

**Table 2:** Blood lead levels for pregnant women, newborns (umbilical cord), and children in Karachi.

	Age ± SD (Children in Months/Women in Years)	n	Arithmetic Mean (±SD)	Median (Interquartile Range)	Range	≥5 µg/dL n (%)	≥10 µg/dL n (%)
Pregnant women	25.24 (3.29)	66	16.18 (8.60)	14.73 (11.21–18.16)	3.33–50.12	65 (98.48)	50 (79.37)
Newborn (umbilical cord)	At birth	61	14.08 (7.95)	12.69 (9.32–15.87)	4.44–42.91	59 (96.97)	41 (67.21)
Male newborn (umbilical cord)	At birth	37	15.54 (9.42)	12.87 (9.35–16.07)	6.37–43.00	37 (100.0)	25 (67.57)
Female newborn (umbilical cord)	At birth	24	11.69 (4.16)	11.81 (8.94–14.38)	4.44–19.10	22 (91.0)	15 (65.22)
Child	25.98 (6.42)	52	21.87 (9.37)	20.11 (14.51–25.36)	8.27–52.14	52 (100.0)	51 (98.08)
Male child	26.72 (6.65)	25	20.67 (8.50)	20.11 (13.97–24.59)	8.27–41.11	25 (100.0)	23 (95.83)
Female child	25.34 (6.24)	27	21.82 (8.45)	18.75 (14.67–25.39)	10.48–47.77	27 (100.0)	27 (100.0)

**Table 3:** Blood lead levels (μg/dL) of study participants (selected families) from Karachi, Pakistan.

Family ID	Pregnant Women	Cord Blood (% of Mother‘s Blood)	Child	Category
A	50.12	43.00 (86)	NA	High
B	49.32	34.52 (70)	52.14	High
C	20.40	16.02 (79)	NA	Medium
D	24.42	18.06 (74)	24.52	Medium
E	12.09	5.54 (46)	14.05	Low
F	11.38	13.42 (118)	25.32	Low
G	11.21	8.94 (80)	11.85	Low
H	11.15	8.93 (80)	18.75	Low

NA: Refused to give consent for blood.

## Appendix A

**Table A1:** Correlation coefficient between lead levels in blood of pregnant women, cord blood, young children, and different sources of exposure in Karachi, Pakistan.

Correlation with Blood Lead Level of Pregnant Women	Spearman’s Rho (ρ)	*p* Value
Cord blood	0.88	<0.001
Young child blood	0.47	<0.001
Pregnant women food	0.29	0.03
Child food	0.32	0.01
House dust	0.38	0.35
Pregnant women water	−0.04	0.76
*Correlation with cord blood*		
Young child blood	0.61	<0.001
Pregnant women food	0.16	0.24
House dust	0.66	0.07
*Correlation with 1-3 years old child’s blood*		
Pregnant women food	0.11	0.46
Young child food	0.38	0.007
Child water	0.006	0.96

**Table A2:** Pb concentration in common food items before and after cooking in different cooking utensils.

	Uncooked (ng/g)	Cooked (ng/g)			
		Steel	Alloy	Iron	Non-Stick
Potato	10.3	9.4	8.9	13.4	10.1
Lentil (daal)	-	34.6	55.1	8.6	9.1
Chicken	-	13.0	19.3	23.0	13.3

**Table A3:** Lead content in petrol and engine lubricant in Karachi, Pakistan.

No.	Type	Pb Concentration (ppm)
1	Petrol	0.083
2	Petrol	0.042
3	Petrol	0.025
4	Petrol	0.018
5	Petrol	0.013
6	Petrol	0.015
7	Lubricant	0.022

**Table A4:** Bioaccessibility of lead from various sources.

Family ID	Mother’s Food	Child’s Food	House Dust	Respirable Dust
A	50%	53%	40%	30%
B	48%	41%	50%	13%
C	131%	92%	52%	23%
D	47%	37%	73%	26%
E	61%	95%	103%	43%
F	65%	80%	93%	54%
G	55%	46%	54%	53%
H	25%	41%	57%	41%
Average	**60%**	**61%**	**65%**	**35%**
